# The association between fast-food consumption with cardiovascular diseases risk factors and kidney function in patients with diabetic nephropathy

**DOI:** 10.34172/jcvtr.2021.42

**Published:** 2021-08-25

**Authors:** Seyyed Reza Sobhani, Mojgan Mortazavi, Mahsa Kazemifar, Leila Azadbakht

**Affiliations:** ^1^Department of Nutrition, Faculty of Medicine, Mashhad University of Medical Sciences, Mashhad, Iran; ^2^Isfahan Kidney Diseases Research Center, Isfahan University of Medical Sciences, Isfahan, Iran; ^3^Department of Community Nutrition, School of Nutritional Sciences and Dietetics, Tehran University of Medical Sciences, Tehran, Iran; ^4^Diabetes Research Center, Endocrinology and Metabolism Clinical Sciences Institute, Tehran University of Medical Sciences, Tehran, Iran; ^5^Food Security Research Center and Department of Community Nutrition, School of Nutrition and Food Science, Isfahan University of Medical Sciences, Isfahan, Iran

**Keywords:** Fast Food, Cardiovascular Diseases, Diabetic Nephropathy

## Abstract

***Introduction:*** Fast food consumption (FFC) has been raised as a risk factor for cardiometabolic outcomes and renal function disorders. The present study aimed to investigate the association between FFC and cardiovascular disease (CVD) risk factors and renal function among patients with diabetic nephropathy (DN).

***Methods:*** This cross-sectional study was conducted among 397 randomly enrolled patients with DN. A validated 168 food items food frequency questionnaire was used for measuring FFC. Weight, waist,height, fasting blood sugar (FBS), hemoglobin A1C (HbA1C), serum creatinine, blood urea nitrogen(BUN), hs-CRP, systolic blood pressure(SBP), diastolic blood pressure (DBP), and lipid profile concentrations were measured. Generalized linear model analysis of covariance was used to compare means of BP, biochemical and anthropometric factors across tertiles of FFC adjusted for potential confounders.

***Results:*** The mean weekly intakes of fast food were 130 ± 60 grams. Patients in the highest compared to the lowest tertiles of FFC were more likely to be overweight and obese, had higher levels of creatinine, SBP, and DBP in the unadjusted model (*P* < 0.05). In the adjusted models, DN patients in the highest vs lowest tertiles of FFC had higher levels of SBP and DBP (*P* = < 0.001).

***Conclusion:*** Higher consumption of fast food is associated with higher levels of both systolic and diastolic blood pressure in DN patients. The present study observed no significant differences between the highest versus the lowest tertiles of FFC for waist, FBS, HbA1C, serum creatinine, BUN, hs-CRP, and lipid profile concentrations.

## Introduction

The main microvascular complexity of diabetes is diabetic nephropathy (DN) and can lead to end-stage renal disease (ESRD), which about 40% of all type 1 diabetic patients suffer it.^1,2^ DN is described by hypertension, advanced albuminuria, a decrease in Glomerular Filtration Rate (GFR), and a great increase in cardiovascular morbidity and mortality that has a significant effect on social wellbeing and economy.^3,4^ It is suggested that 20-30 million people universally are affected by symptomatic DN. Also, about 53% of Iranian type 2 diabetic (T2D) patients develop this condition.^5^ The rate of CardiovascularDisease(CVD) in chronic kidney disease (CKD) patients is between 25% and 60% and the possibility of CVD in these patients is 10 to 20 times higher than the public population.^6,7^

DN, as a progressive disease, has highly complicated pathogenesis in which various cells, molecules, and nutritional factors get involved. ^8^ The reports of some studies show that consumption of omega 6 fatty acids ^9^ and also soy ^10^ can improve complications of DN. However, excess food and salt intake ^11^, as well as deficiency and inadequacy of vitamin D play a crucial role in the pathogenesis of this disorder. ^12^ Overall, a low-protein diet with salt, phosphorus, and potassium restriction in progressive cases, along with suitable control of hypertension, blood glucose, and hyperfiltration, is recommended to postpone the progression of DN.^13^

Poor quality and unhealthy diets mostly contain highly processed food products and low in fresh ingredients.^14^ The food patterns of recent decades show an increase in eating out and gaining energy from sugar and fats. ^15^ Currently, consumption of fast foods, sodium, and sugar becomes more frequent. Therefore, the possibility to meet the recommended nutrients is weak.^16,17^ As a consequence of this developing trend, the prevalence of T2D, risk factors of metabolic syndrome, and the severity of CKD will be incremented which can finally lead to DN. ^18,19^ Clinical and epidemiological studies have suggested that fast food consumption, by higher intakes of energy-dense foods, refined grains, sodium, and soft carbonated beverages, and also lower intakes of fruits, vegetables, milk, vitamin C, and vitamin A can be caused CVD.^14,20,21^

Undoubtedly, fast food consumption has been expanded among Iranians in the last decades. ^22^ Previous studies show that this trend is caused by poor dietary intake and CVD risk factors among Iranian adults ^23^, and also increasing intake of saturated fats, cholesterol, and sodium may lead to renal function disorders. Since, there is no study to evaluate the association between FFC with the CVD risk factors and renal function in patients with DN, this study aimed at examining this relationship among Iranian patients.

## Materials and methods

### Participants

397 patients have included in the study with DN, who were selected from the Alzzahra Hospital, and a nephrology and nutrition clinic in Isfahan, Iran from July 2010 to April 2013. GFR(ml/min) was used as the dependent variable. Glomerular filtration rate (GFR) was calculated on the basis of the following formula: [140 - age (years)] ×[weight (kilograms)]/72 × (serum creatinine).^10^ The formula: N = [(Z _1-a/2_)^2^ S^2^]/d^2^, was used to estimate the sample size. We select 346 subjects using SD = 19, d = 2, a = 0.05 and b = 0.8. DN was affirmed by a nephrologist. For all attendants, total urine protein excretion was between 300 and 1000 mg/d, serum creatinine was between 1 and 2.5 mg/dL, and blood urea nitrogen (BUN) was between 20 and 40 mg/dL. Patients at stage 1 or 2 of DN, FBS _126 mg/dL, taking hypoglycemic agents, or insulin, and proteinuria _300 mg/d be involved. The patients were excluded who were on a certain diet or if their daily energy consumption was < 800 kcal or > 4200 kcal ^24^, or they start a new drug regimen.

### Measurement of fast food consumption

A validated semi-quantitative food frequency questionnaire (FFQ) including 168 food items was used to measure the dietary intake. Food items were selected based on the most intermittently consumed food items in the national food consumption survey in Iran in this questionnaire. As various recipes are used to make foods, the FFQ was relying on food items instead of dishes, such as beans, different meats, oils, and rice. Moreover, the validity and reliability of this questionnaire have been formerly measured in Iran. ^25^ All FFQs were filled out through direct interviews by a trained dietitian. Finally, we transformed portion sizes of food items to grams using household scales.^26^ NUTRITIONIST-IV (version 7.0; Squared Computing, Salem, OR, USA) was used to measure the nutrient and energy intakes. Fast foods were described as the following items: “convenience food” or “ready to eat” foods such as hamburgers, sausage, cheeseburger, other burgers, hot dogs, rusk fish, rusk poultry, French fries, and pizza.^17^ Fast food consumption was presented in g per week.

### Anthropometric assessments

Weight was measured while participants have light clothes and were shoes off using a digital scale and reported to the nearest 100 g. Patients stood in their normal standing position without shoes to determine their height to the nearest 0.5 cm. Waist circumference (WC) was measured at the narrowest area between the last rib and the iliac crest to the nearest 0.5 cm with a tape measure without enforcing any pressure to the body surface and patients were in their light indoor clothes. Body mass index (BMI) was counted as body weight (kg) divided by the square of height in meters (m2).

### Assessment of other variables

The mean of two blood pressure (BP) measurements which were calculated by a standard mercury sphygmomanometer was used for statistical analysis. The subjects were asked to record their physical activity on three separate days to determine the physical activity status. Then their mean was reported to measure their physical activity levels which presented as metabolic equivalents in h/wk. ^27^ A validated Persian questionnaire was used to determine the socioeconomic status (SES) that consisted of questions on family earnings, education level, job status, numbers of family members, and home and car ownership, number of rooms at house, age of furniture they had, and number of trips inside and outside Iran. Subjects were classified into quartiles of SES: weak, moderate, high, and very high.

### Biochemical assessment

Venous blood samples were gathered after an overnight fast. All l biochemical analyses were conducted in 48 hours when blood collection was completed to avoid sample degradation. Commercially available enzymatic indices (Pars Azmoon, Tehran, Iran) which are adaptable to an auto-analyzer system (Selectra E, Vitalab, Holliston, the Netherlands) were used to determine FBS and lipid profile concentrations, including total cholesterol (TC), low-density lipoprotein cholesterol (LDL-C) and triglyceride (TG). The pink reagent kit on a DS5 analyzer was applied to measure HbA1c concentrations from whole blood samples. Serum creatinine and BUN were used along with a colorimetric method and enzymatic colorimetric Bertholet method with a commercially available assay kit. Finally, an ultrasensitive latex-enhanced immunoturbidimetric assay (Randox Laboratory Ltd.Belfast, UK) computed serum levels of hs-CRP.

### Statistical analysis

Characteristics of patients were reported as mean ± standard deviation or number and percentage. Continuous and categorical variables were compared across tertiles of fast food consumption by ANOVA and chi-squared test, respectively. An ANOVA test was used to compare means in all unadjusted models (characteristics and biochemical markers). In adjusted models, we used generalized linear model analysis of covariance to determine statistical significance. Sex, age, energy intake, and physical activity were adjusted in model 1. Medications, phosphor, protein, fat, sodium, cholesterol, and potassium were additionally adjusted in model 2. All mentioned variables in model 2 besides BMI were adjusted in model 3. *P*value < 0.05 was considered significant.

## Results

The mean intakes of fast food were 130 ± 60 grams per week. Table 1shows thecharacteristics of the patients in this study across the tertile categories of FFC. Patients in the highest vs the lowest tertile of FFV were slightly older and more likely to be overweight and obese. There is no significant difference in distributing anthropometric measures, using the medication, socioeconomic status, and abdominal obesity across the tertile categories of fast food consumption.

**Table 1:** Baseline characteristics of patients with diabetic nephropathy by the median of fast food consumption*

**Variables** ^†^	**All patients**	**FFC tertiles**			***P*** ** value**
		**First tertile (n=133)**	**Second tertile (n=132)**	**Third tertile (n=132)**	
Age(yr)	65.54 ± 9.76	66.46 ± 10.20	63.39 ± 9.28^a,b^	66.79 ± 9.47	< 0.01
BMI(kg/m^2^)	23.35 ± 3.53	23.29 ± 3.42	22.95 ± 2.90	23.81 ± 4.14	0.13
WC(cm)	101.96 ± 11.09	102.08 ± 10.12	102.39 ± 12.13	101.42 ± 10.99	0.77
Physical activity (MET.h/wk)	49.3 ± 12.1	47.9 ± 13.0	49.0 ± 11.3	48.6 ± 11.5	0.21
Female (%)	48.4	43.6	59.1	42.4	0.01
Overweight or obese^‡^	29.4	27.5	22.0	44.0	0.01
Abdominal obesity^§^(%)	76.6	74.4	81.8	73.5	0.22
Medications use^¥^(%)	11.8	12.0	10.6	12.9	0.85
SES					
Weak (%)	9.1	10.5	10.6	6.1	
Moderate (%)	31.5	27.8	30.3	36.4	
High (%)	35.5	34.6	37.9	32.6	
Very high (%)	24.4	27.1	21.2	25.0	0.53

BMI, body mass index; MET, metabolic equivalents; SES, socioeconomic status; WC, waistcircumference * Values are mean ± SE unless indicated. Letters indicate differences within a tertile: ^a^vs. other tertiles, *P* <  0.05, ^b^ vs. tertile 1, *P* <  0.05. † P-values were calculated by one way ANOVA for continuous variables and x^2^ for categorical variables. ‡ Overweight or obese: BMI ≥ 25 kg/m^2^ § Abdominally obese: WC ≥ 102 cm for men and WC ≥ 88 cm for women. ¥ Medications included antihypertensives, lipid-lowering agents, antidiabetics, antiacids, hormones, multivitamins and minerals, and anticoagulant agents.

The crude and age with energy-adjusted means intake of nutrients across the tertiles of FFC are presented in Table 2. Higher intakes of fast foods were related to higher levels of energy, protein, fat, carbohydrate, cholesterol, vitamin C, saturated fat, mono fat, oleic fat, poly fat, iron, zinc, magnesium, a-tocopherol, P (phosphorus), K (potassium), Ca (calcium), copper, Na (sodium), B1 (thiamine), B2 (riboflavin), B3 (niacin), B6 (pyridoxine), folate, B12 (cobalamin), and vitamin C in the crude model. All significant relationship in the crude model was unchanged after adjusting for age and total energy intakes in model 1.

**Table 2:** Dietary intakes of patients with diabetic nephropathy by tertiles of Fast Food Consumption*

**Variables**	**FFC tertiles**			***P*** **value**
	**First tertile (n=133)**	**Second tertile (n=132)**	**Third tertile (n=132)**	
Energy (kcal/d)	2189.76 ± 286.54	2150.92 ± 263.75	2238.52 ± 311.32	0.04
Fast food (g/w)	121 ± 35	129 ± 60	135 ± 16	< 0.001
Protein (g/d)	73.44 ± 25.29	87.68 ± 24.86	113.24 ± 30.76	< 0.001
Model 1	73.44 ± 27.19	87.88 ± 27.39	113.05 ± 27.29	< 0.001
Fat (g/d)	60.83 ± 31.76	70.56 ± 37.42	87.71 ± 42.38	< 0.001
Model 1	60.75 ± 37.57	70.82 ± 37.58	87.53 ± 37.70	< 0.001
Carbohydrate (g/d)	312.69 ± 117.87	313.03 ± 84.34	353.57 ± 98.34	< 0.01
Model 1	313.02 ± 101.48	313.20 ± 102.22	353.07 ± 101.82	0.001
Cholesterol (mg/d)	147.82 ± 65.40	187.61 ± 66.37	257.27 ± 91.00	< 0.001
Model 1	147.81 ± 75.50	187.68 ± 76.06	257.23 ± 75.75	< 0.001
Vitamin C (mg/d)	158.51 ± 98.77	151.32 ± 98.44	190.07 ± 125.33	0.01
Model 1	158.75 ± 108.00	152.93 ± 108.29	188.22 ± 108.35	0.02
Saturated Fat (g/d)	19.22 ± 12.52	21.61 ± 13.24	27.65 ± 16.21	< 0.001
Model 1	19.26 ± 14.13	21.56 ± 14.18	27.67 ± 14.18	< 0.001
Mono fat	18.10 ± 10.68	22.01 ± 12.09	28.32 ± 15.77	< 0.001
Model 1	18.12 ± 13.08	22.03 ± 13.18	28.29 ± 13.12	< 0.001
Oleic fat	14.22 ± 9.52	17.47 ± 11.23	22.95 ± 14.80	< 0.001
Model 1	14.22 ± 12.10	17.52 ± 12.19	22.90 ± 12.14	< 0.001
Poly Fat (g/d)	14.66 ± 7.21	17.59 ± 12.07	20.79 ± 13.47	< 0.001
Model 1	14.62 ± 11.28	17.69 ± 11.35	20.74 ± 11.32	< 0.001
iron (mg/d)	12.36 ± 6.00	13.31 ± 4.72	15.95 ± 4.73	< 0.001
Model 1	12.34 ± 5.19	13.41 ± 5.23	12.86 ± 5.20	< 0.001
Zinc (mg/d)	7.85 ± 2.52	8.98 ± 3.25	10.69 ± 3.23	< 0.001
Model 1	7.85 ± 3.03	9.01 ± 3.06	10.67 ± 3.04	< 0.001
Magnesium (mg/d)	248.99 ± 97.25	306.70 ± 113.91	363.26 ± 114.44	< 0.001
Model 1	284.75 ± 109.11	308.02 ± 109.90	362.19 ± 109.47	< 0.001
Manganese (mg/d)	3.23 ± 1.26	3.58 ± 1.53	3.46 ± 1.05	0.07
Model 1	3.23 ± 1.30	3.59 ± 1.31	3.46 ± 1.30	0.08
Vitamin A (mcg/d)	1087.22 ± 1617.48	902.05 ± 552.88	1039.36 ± 475.01	0.31
Model 1	1083.87 ± 1023.73	926.46 ± 1031.21	1018.33 ± 1027.12	0.46
Beta-carotene (UA/d)	860.84 ± 634.93	810.68 ± 640.72	880.26 ± 514.77	0.62
Model 1	856.01 ± 597.66	828.10 ± 602.03	867.72 ± 599.64	0.86
A-Tocopherol (mg/d)	5.87 ± 2.32	6.64 ± 3.66	7.17 ± 2.90	< 0.01
Model 1	5.87 ± 3.02	6.65 ± 3.04	7.17 ± 3.03	< 0.01
Dietary fiber (g/d)	16.72 ± 7.54	17.68 ± 13.66	19.57 ± 8.07	0.07
Model 1	16.73 ± 10.18	17.71 ± 10.25	19.53 ± 10.21	0.08
Soluble Fiber (g/d)	0.73 ± 0.62	0.66 ± 0.32	0.77 ± 0.45	0.21
Model 1	0.73 ± 0.48	0.67 ± 0.48	0.77 ± 0.48	0.28
Crude Fiber (g/d)	5.11 ± 3.44	5.30 ± 4.44	5.98 ± 2.31	0.11
Model 1	5.12 ± 3.52	5.30 ± 3.55	5.98 ± 3.54	0.12
P (mg/d)	1395.04 ± 493.85	1529.23 ± 565.81	1859.05 ± 586.74	< 0.001
Model 1	1393.77 ± 552.23	1533.57 ± 556.26	1855.99 ± 554.05	< 0.001
K (mg/d)	3133.36 ± 1352.20	3319.62 ± 1198.52	3860.54 ± 1273.22	< 0.001
Model 1	3133.68 ± 1277.70	3336.46 ± 1287.03	3843.40 ± 1281.92	< 0.001
Ca (mg/d)	1066.87 ± 447.02	1107.73 ± 509.97	1247.36 ± 507.14	< 0.01
Model 1	1065.99 ± 490.24	1113.30 ± 493.81	1242.69 ± 491.86	0.01
copper (mg/d)	1.42 ± 0.48	1.61 ± 0.65	1.81 ± 0.66	< 0.001
Model 1	1.43 ± 0.60	1.61 ± 0.61	1.81 ± 0.61	< 0.001
Na (mg/d)	6670.79 ± 7164.33	5360.98 ± 4231.84	7778.23 ± 8679.32	0.02
Model 1	6717.35 ± 6881.91	5457.61 ± 6932.14	7634.70 ± 6904.62	0.04
B1 (mg/d)	1.40 ± 0.60	1.46 ± 0.62	1.64 ± 0.45	< 0.001
Model 1	1.40 ± 0.57	1.47 ± 0.56	1.64 ± 0.56	0.003
B2 (mg/d)	2.13 ± 0.88	2.29 ± 0.83	2.58 ± 0.83	< 0.001
Model 1	2.13 ± 0.85	2.31 ± 0.86	2.57 ± 0.86	< 0.001
B3 (mg/d)	16.24 ± 7.86	19.72 ± 8.37	26.21 ± 8.84	< 0.001
Model 1	16.27 ± 8.40	19.57 ± 8.46	26.16 ± 8.42	< 0.001
B6 (mg/d)	1.45 ± 0.69	1.58 ± 0.58	2.09 ± 0.66	< 0.001
Model 1	1.45 ± 0.65	1.60 ± 0.65	2.07 ± 0.64	< 0.001
Folate (mcg/d)	302.01 ± 150.57	302.11 ± 145.54	343.42 ± 117.55	0.02
Model 1	301.33 ± 138.44	305.50 ± 139.44	340.72 ± 138.89	0.04
B12 (mcg/d)	3.67 ± 4.11	4.65 ± 2.79	6.02 ± 4.69	< 0.001
Model 1	3.68 ± 3.94	4.68 ± 3.98	5.98 ± 3.96	< 0.001

DN, diabetic neuropathy * Values are mean ± SD. † P-values were calculated by ANOVA in all unadjusted models and generalized linear model analysis of covariance in adjusted model that nutrients were adjusted for age and total energy intake.

Table 3 shows biochemical markers, BP, and waist circumference across the tertile categories of fast food consumption in four models. Crude shows the unadjusted model. As mentioned above, Model 1 is adjusted for sex, age, total calorie intake, and physical activity. Model 2 is additionally adjusted for medications, phosphor, protein, fat, sodium, cholesterol, and potassium. Model 3 is adjusted for all mentioned variables in model 2 besides BMI. Patients in the highest versus lowest tertile of FFC have higher levels of creatinine, SBP, DBP, and lower levels of total cholesterol. This relationship was significant between SBP and DBP, even after additional control for potential confounding variables in model 1, model 2, and model 3.

**Table 3:** The crude and adjusted mean values of biochemical factors, blood pressure, and waist circumference across tertiles of fast-food consumption among the study population

**Variables**	**FFC tertiles**			***P*** **value**
	**First tertile (n=133)**	**Second tertile (n=132)**	**Third tertile (n=132)**	
Fasting blood sugar (mg/Dl)				
crude	128.76 ± 13.71	128.73 ± 15.35	131.08 ± 15.74	0.34
Model 1	128.43 ± 13.90	130.10 ± 14.10	129.99 ± 13.81	0.55
Model 2	128.90 ± 15.73	130.48 ± 13.94	129.14 ± 16.13	0.62
Model 3	128.84 ± 15.76	130.55 ± 13.99	129.13 ± 16.14	0.58
HbA_1C_ (%)				
crude	8.00 ± 13.77	6.28 ± 0.53	6.30 ± 0.45	0.13
Model 1	8.01 ± 7.98	6.48 ± 8.10	6.02 ± 7.93	0.08
Model 2	7.93 ± 9.18	6.38 ± 8.13	6.29 ± 9.41	0.30
Model 3	7.95 ± 9.20	6.35 ± 8.17	6.29 ± 9.42	0.25
Total cholesterol (mg/dL)				
crude	271.24 ± 21.67	273.19 ± 16.64	265.37 ± 28.66	0.02
Model 1	272.05 ± 22.84	272.51 ± 23.17	265.11 ± 22.70	0.01
Model 2	268.64 ± 26.15	272.18 ± 23.18	268.89 ± 26.82	0.40
Model 3	268.70 ± 26.22	272.10 ± 23.28	268.90 ± 26.84	0.42
Triglyceride (mg/dL)				
crude	255.10 ± 35.32	253.62 ± 29.87	246.00 ± 36.95	0.07
Model 1	254.18 ± 33.05	255.05 ± 33.54	245.41 ± 32.85	0.03
Model 2	252.45 ± 37.89	254.96 ± 37.57	247.25 ± 38.83	0.25
Model 3	252.66 ± 37.92	254.70 ± 33.66	247.31 ± 38.82	0.28
LDL-C (mg/dL)				
crude	133.44 ± 20.42	135.70 ± 20.98	132.10 ± 19.57	0.35
Model 1	133.49 ± 20.67	136.17 ± 20.96	131.82 ± 20.54	0.24
Model 2	134.77 ± 23.75	136.08 ± 21.05	130.63 ± 24.34	0.17
Model 3	134.59 ± 23.74	136.30 ± 21.07	130.58 ± 24.30	0.14
Creatinine (mg/dL)				
crude	1.91 ± 0.24	2.00 ± 0.23	1.97 ± 0.27	0.01
Model 1	1.91 ± 0.25	2.00 ± 0.25	1.97 ± 0.24	0.01
Model 2	1.92 ± 0.28	2.00 ± 0.25	1.97 ± 0.29	0.06
Model 3	1.92 ± 0.29	2.00 ± 0.25	1.97 ± 0.29	0.07
BUN (mg/dL)				
crude	19.22 ± 2.49	19.61 ± 2.63	19.28 ± 2.68	0.41
Model 1	19.16 ± 2.60	19.60 ± 2.63	19.32 ± 2.58	0.39
Model 2	19.34 ± 2.98	19.63 ± 2.63	19.10 ± 3.04	0.32
Model 3	19.34 ± 2.99	19.63 ± 2.64	19.10 ± 3.05	0.32
hs_CRP (mg/L)				
crude	1.59 ± 0.33	1.55 ± 0.28	1.61 ± 0.32	0.25
Model 1	1.59 ± 0.31	1.54 ± 0.31	1.62 ± 0.31	0.08
Model 2	1.60 ± 0.36	1.53 ± 0.29	1.62 ± 0.37	0.07
Model 3	1.60 ± 0.36	1.53 ± 0.31	1.62 ± 0.37	0.08
Systolic blood pressure (mm Hg)				
crude	104.53 ± 9.45	101.06 ± 12.81	107.28 ± 7.72	< 0.001
Model 1	104.69 ± 10.27	101.08 ± 10.42	107.30 ± 10.20	< 0.001
Model 2	105.10 ± 11.84	101.15 ± 10.50	106.82 ± 12.14	< 0.001
Model 3	105.04 ± 11.87	101.22 ± 10.54	106.81 ± 12.15	< 0.001
Diastolic blood pressure (mm Hg)				
crude	72.77 ± 8.75	71.96 ± 9.11	75.68 ± 9.22	< 0.01
Model 1	72.64 ± 8.90	72.01 ± 9.03	75.97 ± 8.85	< 0.01
Model 2	72.06 ± 10.27	71.93 ± 9.09	76.64 ± 10.53	< 0.01
Model 3	72.10 ± 10.29	71.88 ± 9.13	76.65 ± 10.53	< 0.001
Waist circumference (cm)				
crude	69.51 ± 7.23	70.93 ± 8.03	70.64 ± 9.02	0.33
Model 1	69.68 ± 8.11	70.72 ± 8.24	70.53 ± 8.10	0.55
Model 2	71.20 ± 9.01	71.00 ± 7.98	68.71 ± 9.24	0.08
Model 3	71.31 ± 8.95	70.87 ± 7.95	68.74 ± 9.17	0.08

BMI, body mass index; BUN, blood urea nitrogen; DN, diabetic neuropathy; Hb, hemoglobin; LDL-C, low-density lipoprotein cholesterol; * * *P* values were calculated by ANOVA in all unadjusted models and generalized linear model analysis of covariance in adjusted models,. Sex, age, energy intake, and physical activity were adjusted in model 1. Medications, phosphor, protein, fat, sodium, cholesterol, and potassium were additionally adjusted in model 2. All mentioned variables in model 2 besides BMI were adjusted in model 3.

## Discussion

This study showed a direct relationship between fast food consumption and both systolic and diastolic blood pressure. Secondly, there is a positive relationship between FFC and intake of energy, protein, fat, carbohydrate, cholesterol, saturated fat, P, K, copper, and Na. Unexpectedly, this relationship was also observed for vitamin C, mono fat, oleic fat, poly fat, iron, zinc, magnesium, a-tocopherol, Ca, B1, B2, B3, B6, folate, and B12. Moreover, those patients who consumed higher amounts of fast food were more likely to be overweight or obese.

Hypertension can evolve a series of complications, like kidney and CVD ^28^ and this is a known risk factor for the failure of kidney function ^29^. Higher fast food consumption decreases dietary quality, which results in the incidence of metabolic disorders such as hypertension.^30^ Despite a study that reported that western pattern diets with high consumption of fast foods were highly related to an increased risk of hypertension among Iranian women ^31^, to date, no study has shown the relationship between fast food consumption and hypertension in patients with DN. In this study, high intake of sodium may affect the significant positive relationship between hypertension and fast food consumption. The amount of sodium in fast foods is often higher than recommended level, as is reported by some common fast food meals, which shows a salt content of about 4.4 to 9.1 gr per meal. ^32^ Insulin resistance and metabolic syndrome markers can be aggravating by a high salt diet besides increasing blood pressure. Most studies approved the relationship between fast food consumption and sodium intake and our findings confirmed this relationship again. A meta-analysis of randomized controlled trials reported that a decrease in sodium intake by 50 mmol per day for at least four weeks could reduce systolic blood pressure by a mean of 4.0 mm Hg and diastolic blood pressure by a mean of 2.5 mm Hg in hypertensive patients and also led to a decline in systolic blood pressure by a mean of 2.0 mm Hg and diastolic blood pressure by a mean of 1.0 mm Hg in normotensive subjects. ^33^ According to Guyton’s suggestion, this protocol can increase blood pressure through volume expansion and also cardiac output first, secondary effects resistance vessels by an autoregulatory mechanism. ^34^

As mentioned above, those who consumed more fast food were more likely to be overweight or obese. This fınding is similar to Ginny Garcia et all study which suggested that fast food consumption among the behavioral factors, has the largest effect on higher levels of obesity.^35^ Moreover, a systematic review supported the independent role of fast food consumption in accelerating rates of weight gain or obesity by increasing caloric intake. In addition, to the increase in fast food consumption, many other aspects of this type of food are suspected of its relationship with overweight and obesity. Specifically, fast foods have loads of energy, higher glycemic load, and also excess portion sizes besides poor micronutrients and lower fiber which lead to intake of daily energy requirements more than the recommended level. ^36^

The amount of trans fats in some of the most well-known fast foods were up to 24g/serving ^37^. The high content of artificially produced trans fatty acids in fast foods is a significant reason to gain weight, abdominal fat aggregation, the progress of insulin resistance, and cardiovascular events.^38^ Moreover, according to our observation, increasing overweight and obesity chances can be caused by higher intakes of energy, fat, carbohydrate, cholesterol, and saturated fat, which emerged from greater consumption of fast foods. Bowman et alreported that both males and females who consumed fast food had higher intakes of energy, total fat, saturated fat, carbohydrate, added sugars, and protein, which is similar to our findings, and lower intakes of vitamin A, carotenes, vitamin C, calcium and magnesium density that is not supported by our findings.^39^ Our findings in this study are similar to findings reported by the study of FFC which fast-food intake was related to higher use of calcium among adults.^40^ This may reflect the tendency of using dairy beverages and vegetables besides the fast foods in Iran.

This study includes several limitations. First of all, although various confounders had been controlled in our analysis, residual confounding cannot be excluded due to unknown or unmeasured confounders. Moreover, there was no evidence of a causal relationship between fast food consumption and other factors provided by the cross-sectional study design. Thirdly, it should be mentioned that Food Frequency Questionnaire (FFQ) has some limitations to measure dietary intakes (such as measurement errors). Despite all these limitations, to our knowledge, this was the first study to measure the relationship between fast food consumption and CVD risk factor besides the renal function in DN patients.

## Conclusion

In conclusion, we identified that fast food consumption was positively related to both systolic and diastolic blood pressure. Also, those who consumed more fast food were more likely to be overweight or obese.Fast food consumption is related to poor dietary intake and as a result, adverse complications such as higher levels of blood pressure, particularly in patients with diabetic nephropathy, so that strongly recommended that DN patients avoid using fast foods or consume at least amounts of dietary fast foods.

## Acknowledgments

Not applicable.

## Competing interest

The authors declare that they have no competing interests.

## Ethical Approval

The Medical Research Ethics Committee of Isfahan University of Medical Sciences confirmed our study with 5556 ethical code. An informed written agreement was taken from all patients to show their satisfaction to participate in the study.

## Funding

Funding was provided by the Isfahan University of Medical Sciences.
